# Triaging HPV-positive, cytology-negative cervical cancer screening results with extended HPV genotyping and p16^INK4a^ immunostaining in China

**DOI:** 10.1186/s12879-021-06109-4

**Published:** 2021-04-30

**Authors:** Fangbin Song, Peisha Yan, Xia Huang, Chun Wang, Xinfeng Qu, Hui Du, Ruifang Wu

**Affiliations:** 1 Department of Obstetrics and Gynecology, Peking University Shenzhen Hospital, 1120 Lianhua Road, Shenzhen, Guangdong 518036 P. R. China; 2 Institute of Obstetrics and Gynecology, Shenzhen PKU-HKUST Medical Center, Shenzhen, Guangdong 518036 P. R. China; 3 Shenzhen Key Laboratory on Technology for Early Diagnosis of Major Gynecological Diseases, Shenzhen, Guangdong 518036 P. R. China; 4 Sanming Project of Medicine in Shenzhen, Peking University Shenzhen Hospital, Shenzhen, Guangdong 518036 P. R. China

**Keywords:** Human papillomavirus, Cervical cancer screening, Cervical intraepithelial neoplasia, HPV genotyping, p16 immunocytochemistry

## Abstract

**Background:**

Self-sampling for human papillomavirus (HPV) testing is a feasible option to improve the cervical screening coverage. However, an ideal triage method for HPV-positive self-samples does not yet exist. The aim of this study was to explore the utility of HPV genotyping and p16^INK4a^ immunostaining (p16) in triaging HPV-positive self-samples, focusing on HPV-positive, cytology-negative (HPCN) women.

**Methods:**

A total of 73,699 women were screened in a cervical screening project in China via SeqHPV assay on self-samples. HPV-positive women were called-back and collected cervical sample for p16 immunostaining and liquid-based cytology, those who met any result of HPV16/18+ or visual inspection with acetic acid (VIA) + or p16+ were referred for colposcopy, and HPCN women with adequate data on p16 and pathology were analyzed. A triage strategy was considered acceptable if the negative predictive value (NPV) for cervical intraepithelial neoplasia 3 or worse (CIN3+) was 98% or more, combined with an improvement of sensitivity and specificity for CIN2+/CIN3+ in reference to the comparator, being HPV16/18 + .

**Results:**

A total of 2731 HPCN women aged 30–64 years were enrolled, 136 (5.0%) CIN2+ and 53 (1.9%) CIN3+ were detected. Five triage strategies met the criteria: p16+; HPV16/33+; ‘HPV16+ or HPV33/58/31/35+&p16+’; ‘HPV16/33+ or HPV58/31/35+&p16+’; HPV16/18/31/33/45/52/58 + & p16+. These strategies required less or similar colposcopy referrals, and less colposcopies to detected one case of CIN2+/CIN3+, achieving favorable false positive (negative) rates to the comparator. Among them, p16 staining detected 83.1% (79.2%) of underlying CIN2 + (CIN3+) in HPCN women. Moreover, three triage strategies were favorable in sensitivity and/or specificity to the ‘HPV16/33+’ strategy: p16+; ‘HPV16+ or HPV33/58/31/35 + &p16+’; HPV16/18/31/33/45/52/58 + &p16 + .

**Conclusions:**

Genotyping for HPV16/33 could be utilized to optimize the management of HPCN women. Moreover, p16 immunostaining, either alone or combined with extended genotypes, is more effective than HPV genotypes alone in the triage of HPCN women.

**Supplementary Information:**

The online version contains supplementary material available at 10.1186/s12879-021-06109-4.

## Background

High-risk human papillomavirus (hrHPV) is assumed to be an indispensable cause of cervical cancers [1], thus HPV testing is being incorporated into cervical screening to improve cervical cancer prevention in an increasing number of countries [2, 3]. However, the majority of HPV infections are transient and quickly cleared [2, 4]. HPV-positive women with abnormal cytology require a referral to colposcopy, but the management of HPV-positive, cytology-negative (HPCN) women remains controversial [5]. HPCN is the most common screen-positive result encountered in clinical practice adopting whether HPV-based or HPV/cytology cotesting screening [4, 6–8], thus its management will pose substantial impacts on screening workload and costs. Although the average risks of cervical intraepithelial neoplasia 3 or worse (CIN3+) among HPCN women were relatively low (2.1%/4.3%/6.4% at 1/3/5 years, respectively) [9], given that the cytology is affected by many factors such as slides production and the highly subjective nature of slides interpretation, cervical high-grade lesions missed by cytology often occur in HPV-positive women [10], particularly in countries with limited well-trained cytologists like China.

The optimal management of HPCN women remains a huge challenge. Triage options for HPCN women including immediate referral to colposcopy, repeat cytology/HPV within 12 months, or triaging with genotypes, viral loads, HPV E6/E7 protein, or other biomarkers [11]. However, immediate referral of HPCN women causes substantial colposcopy burden and overtreatment, while short-term repeat tests can produce anxiety for women involved and entail appreciable loss to follow-up [11]. The 2012 ASCCP guidelines recommend that HPCN women infected with the most oncogenic genotype-16/18 should be referred for immediate colposcopy, whereas those positive for other hrHPV be followed-up in one-year [5]. The 2019 ASCCP guidelines recommend that any set of results predicting an underlying immediate risk of ≥4.0% for CIN3+ are referred to colposcopy [12].

HPV genotype is a potent predictor of the risk for oncogenic progression [9]. However, type-distribution varies from regions and ethics, for instance, HPV58/52 has found to be the most prevalent types and a high contribution of HPV58 to cervical cancer has been reported in Asia [13, 14]. Additionally, genotype-HPV16/18 is becoming rarer in the vaccine era, and genotyping solely for HPV16/18 would miss partial underlying CIN2+ caused by extended genotypes. Several evidences suggest that HPV31/33/58 pose disease risk above HPV18 [6, 10, 15, 16], it’s questionable whether alternative triage scheme developed by extended genotypes could improve the management of HPCN women.

Genotypes can stratify risk for cervical diseases among HPCN results [15], but cannot distinguish between transient and persistent infections. p16^INK4a^(p16) immunostaining indicates HPV-induced cell-cycle deregulation and predicts the transformation process and future progression of HPV infection [17], thus may have the potential to identify underlying lesions missed by cytology in HPV-positive women. Currently, p16 immunostaining is widely utilized in clinical practice to increase the accuracy of histologic diagnosis, whereas it’s limited to cervical biopsy or excision samples; both are invasive procedures [18]. p16 immunostaining on cervical brushing sample from a minimally invasive procedure is clinically relevant.

Due to the convenience and easy-to-operate of HPV-based self-sampling, it could serve as a tool to increase the coverage of cervical screening in China. Moreover, both our and other clinical trials have confirmed that PCR-based HPV testing has similar accuracy on self-samples and clinician-samples [7, 19–21]. Based on a large population-based screening program using SeqHPV assay on self-samples, we aimed to explore whether extended genotypes and p16 immunocytochemistry, alone or combined, could optimize the management of HPCN women.

## Materials and methods

### Study design, participants and procedures

**Fig. 1:**
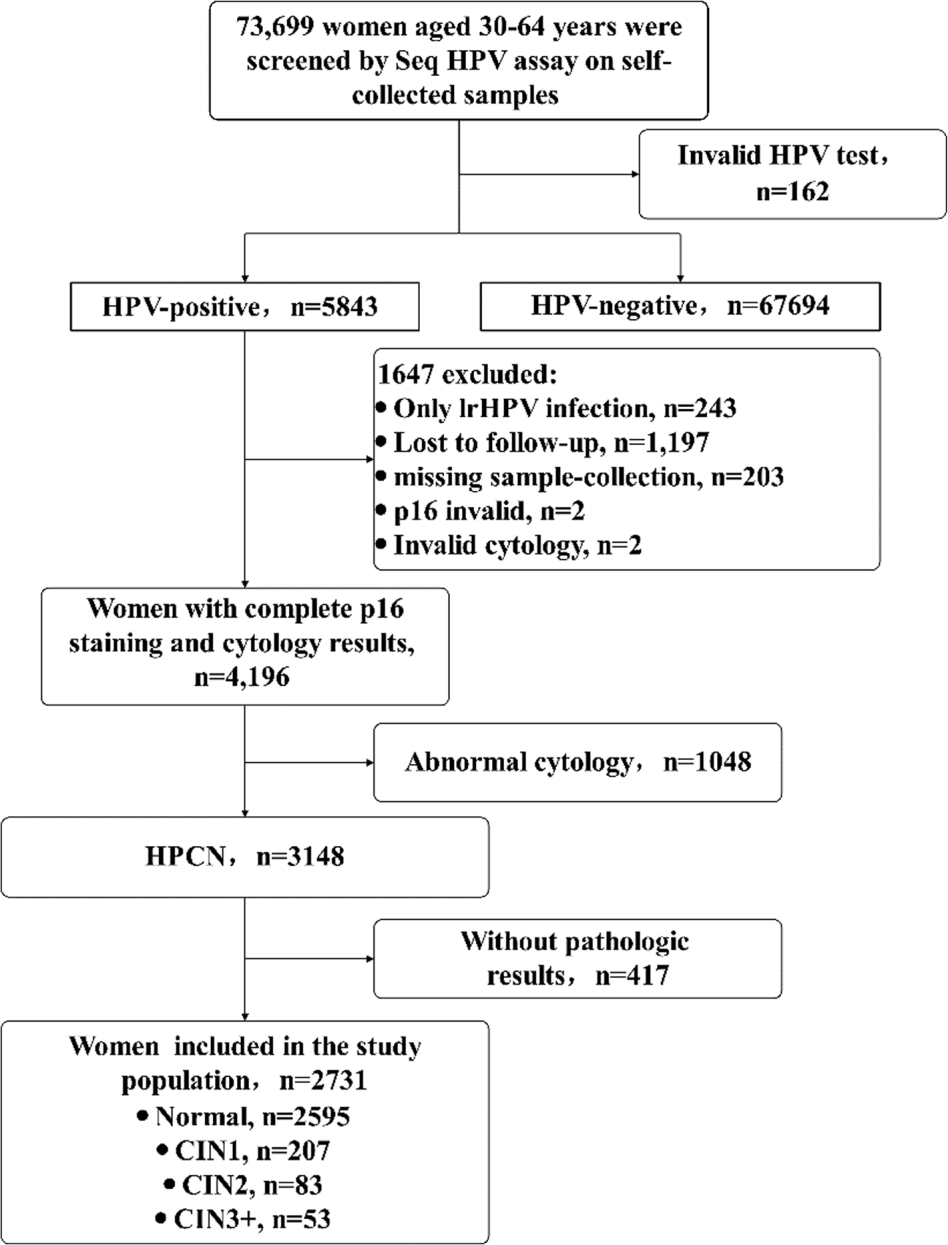
Selection process of study population. Women with HPV-positive, cytology-negative results and valid pathologic results were included in the analysis. lrHPV = low-risk HPV

This prospective observational study was nested into a large population-based cervical cancer screening program from Nov 2018 to Dec 2019, which was organized by a prefecture city in Henan Province, China. No large-scale cervical screening program was organized previously in the city. A total of 73,699 non-pregnant women aged 30–64 years, without history of cervical neoplasia or cancer, without uterine or cervical resection were screened via SeqHPV assay as the primary screening on self-collected samples. Women with HPV-negative result were advised to regular screening by HPV assay after 3 years. While HPV-positive women were called-back for collection of a cervical sample by local gynecologists prior to their colposcopy or visual inspection under acetic acid (VIA). The cervical sample was used for p16 immunostaining and liquid-based cytology (LBC), and LBC was used for research purpose but not for patient care (Fig. S1). HPV-positive women were referred for colposcopy/biopsy if they were (a) HPV16/18+; (b) other HPV+ and VIA+; (c) other HPV+, VIA negative but p16+. Women with complete data on p16 staining, LBC, genotypes and pathology were analyzed (Fig. 1). The study protocol and the digital informed consent was approved by the Ethics Committee from Peking University Shenzhen Hospital (No.2018035). All methods were carried out in accordance with the rules of the Declaration of Helsinki. The informed consent was obtained from every participant.

### Self-sampling and genotyping

After registration for participation via the mobile device, each participant offered a cervicovaginal specimen collected with a nylon conical brush by herself, following user instructions on a printed guide. Cervicovaginal samples were collected by participants themselves and placed on the solid media transport card (iFTA, GE Healthcare, United Kingdom). Subsequently, self-collected samples were prepared for Seq HPV assay (BGI Shenzhen, Shenzhen, China), an HPV genotyping assay using multiplex PCR and next generation sequencing [22]. Seq HPV assay has been previously validated in two large clinical trials [21, 22], and acquired approval from the China Food and Drug Administration (CFDA). The assay separately detects 14 types of hrHPV (16/18/31/33/35/39/45/51/52/56/58/59/66/68) and two types of low-risk HPV (lrHPV, HPV6/11).

### p16^INK4a^ immunostaining and LBC

When HPV-positive women returned for their secondary visit, the local clinicians, who were trained by the research team, used a cyto-brush to collect a cervical specimen. The sample was placed in the preservation solution (Shenzhen Senying Biotechnology, China). Thin-layer cell slides were prepared for p16^INK4a^ immunostaining, which was carried out by PathCIN®p16^INK4a^ automatic immunocytochemistry system (Shenzhen Senying Biotechnology, China) via using a p16^INK4a^ monoclonal antibody (clone sy-a01) at a dilution of 1:400. Moreover, ten samples can be detected on one slide at the same time, which saves cost and qualifies p16 immunostaining with a high-throughput capability. The residual specimens after p16 staining were used for LBC test. p16 staining and LBC slides were independently read by two senior cytologists in the Peking University Shenzhen Hospital blinded to other data except HPV positivity, and discordant results were resolved via consensus review. One or more cervical epithelial cell with nuclear and/or cytoplasmic stained brownish-yellow were classified as positive regardless of their morphology [23]. Slides without stained-cell were defined as negative (Fig. S2). The LBC slides were interpreted by the Bethesda system (TBS) criteria [24], and atypical squamous cells of undetermined significance or worse (≥ASC-US) was defined as abnormal cytology.

### Colposcopy and biopsy

Women that underwent colposcopy received direct and random biopsies by quadrant [25], or endocervical curettage (ECC) when colposcopy was unsatisfactory to improve disease ascertainment. Specifically, random biopsies were taken at 2/5/8/11 o’clock for HPV-positive women without visible lesions. Multiple biopsies were taken at the VIA-positive site(s) plus the opposite quadrant of the transformation-zone for patients with visible lesion(s). Colposcopies were scheduled to be completed within 3 months (actually at 2–6 months) after primary screening. Histologic results were interpreted on the basis of the cervical intraepithelial neoplasia (CIN) histologic grading system including: normal, LSIL (CIN1), HSIL (CIN2/3), and cervical cancers. Women had a negative result of cytology, p16 staining and VIA were deemed pathologically normal.

### Evaluated triage strategies (Tables 1 & 2)

The following triage algorithms were evaluated:

**Table 1:** Clinical performance of p16 immunostaining and extended genotyping, combined or alone, for detection of CIN2+ (*n* = 136) in HPV-positive, cytology-negative women

Triage strategies	Sensitivity, % (95% CI) ^**a**^	Specificity, % (95% CI) ^**a**^	PPV, % (95% CI)	NPV, % (95% CI)	Relative sensitivity ^**b**^	Relative specificity ^**b**^
**Single test**						
1.p16+ ^c^	83.1 (75.7–89.0) **††	77.5 (75.8–79.1) ***††	16.2 (13.6–19.2)	98.9 (98.2–99.3)	1.22	1.12
2.HPV16+	62.5 (53.8–70.6) **	79.2 (77.6–80.8) ***	13.6 (11.1–16.6)	97.6 (96.8–98.2)	0.91	1.14
3.HPV16/33+	67.6 (59.1–75.4)	74.4 (72.7–76.1) ***	12.2 (10.0–14.8)	97.8 (97.0–98.4)	0.99	1.07
4.HPV16/33/58+	77.9 (70.0–84.6) *	61.9 (60.0–63.8) ***	9.7 (8.0–11.6)	98.2 (97.4–98.7)	1.14	0.89
5.HPV16/33/58/31+	81.6 (74.1–87.7) **	56.5 (54.6–58.4) ***	9.0 (7.4–10.7)	98.3 (97.5–98.9)	1.19	0.81
6.HPV16/33/58/31/35+	83.1 (75.7–89.0) **	54.2 (52.3–56.2) ***	8.7 (7.2–10.4)	98.4 (97.6–99.0)	1.22	0.78
*7.HPV16/18+ (comparator)*	68.4 (59.9–76.1)	69.4 (67.6–71.1)	10.5 (8.6–12.7)	97.7 (96.8–98.3)	1.00	1.00
8.HPV16/18/31/33/45/52/58+	91.9 (86.0–95.9) ***	32.2 (30.4–34.0) ***	6.6 (5.6–7.9)	98.7 (97.6–99.3)	1.34	0.46
**HPV types + or p16+**						
9.HPV16+ or p16+	93.4 (87.8–96.9) ***	64.5 (62.6–66.3) ***	12.1 (10.2–14.3)	99.5 (98.9–99.7)	1.37	0.93
10.HPV16/33+ or p16+	94.1 (88.7–97.4) ***	60.6 (58.7–62.5) ***	11.1 (9.4–13.1)	99.5 (99.0–99.8)	1.38	0.87
11.HPV16/33/58+ or p16+	97.1 (92.6–99.2) ***	50.9 (49.0–52.8) ***	9.4 (7.9–11.1)	99.7 (99.2–99.9)	1.42	0.73
12.HPV16/33/58/31+ or p16+	97.1 (92.6–99.2) ***	46.4 (44.5–48.4) ***	8.7 (7.3–10.2)	99.7 (99.1–99.9)	1.42	0.67
13.HPV16/33/58/31/35+ or p16+	97.1 (92.6–99.2) ***	44.6 (42.7–46.5) ***	8.4 (7.1–9.9)	99.7 (99.1–99.9)	1.42	0.64
14.HPV16/18+ or p16+	94.9 (89.7–97.9) ***	58.0 (56.1–59.9) ***	10.6 (8.9–12.5)	99.5 (99.0–99.8)	1.39	0.84
15.HPV16/18/31/33/45/52/58+ or p16+	97.8 (93.7–99.5) ***	27.8 (26.1–29.6) ***	6.6 (5.6–7.8)	99.6 (98.7–99.9)	1.43	0.40
**HPV types + & p16+**						
16.HPV16+ or HPV33/58/31/35 + &p16+ ^c^	79.4 (71.6–85.9) **†††	74.1 (72.4–75.8) ***†	13.8 (11.5–16.5)	98.6 (97.9–99.0)	1.16	1.07
17.HPV16/33+ or HPV58/31/35 + &p16+ ^c^	80.1 (72.4–86.5) **†††	70.3 (68.5–72.0) †††	12.4 (10.3–14.8)	98.5 (97.9–99.0)	1.17	1.01
18.HPV16/33/58/31/35 + &p16+	69.1 (60.6–76.8)	87.1 (85.7–88.4) ***	21.9 (18.1–26.2)	98.2 (97.5–98.7)	1.01	1.26
19.HPV16/18/31/33/45/52/58 + &p16+ ^c^	77.2 (69.2–84.0) †	81.8 (80.3–83.3) ***†††	18.2 (15.2–21.7)	98.6 (97.9–99.0)	1.13	1.18

*Abbreviations*: *CI* confidence interval, *NPV* negative predictive value, *PPV* positive predictive value

^a^*P*-values for accuracy of the evaluated assay vs the ‘HPV16/18+’ strategy, * < 0.05, ** < 0.01, *** < 0.001

^b^Relative sensitivity or specificity of evaluated triage strategies relative to the ‘HPV16/18+’ triage

^c^*P*-values for accuracy of the evaluated assay vs the ‘HPV16/33+’ strategy, † > 0.05; †† *p* < 0.01; ††† < 0.001

**Table 2:** Clinical performance of p16 immunostaining and extended genotyping, combined or alone, for detection of CIN3+ (*n* = 53) in HPV-positive, cytology-negative women

Triage strategies	Sensitivity, % (95% CI) ^**a**^	Specificity, % (95% CI) ^**a**^	PPV, % (95% CI)	NPV, % (95% CI)	Relative sensitivity ^**b**^	Relative specificity ^**b**^
**Single test**						
1.p16+ ^c^	79.2 (65.9–89.2) †	75.5 (73.8–77.1) ***†	6.0 (4.4–8.1)	99.5 (99.0–99.7)	0.86	1.10
2.HPV16+	86.8 (74.7–94.5)	78.4 (76.8–80.0) ***	7.4 (5.5–9.8)	99.7 (99.3–99.9)	0.94	1.14
3.HPV16/33+	90.6 (79.3–96.9)	73.6 (71.8–75.2) ***	6.3 (4.8–8.4)	99.7 (99.4–99.9)	0.98	1.07
4.HPV16/33/58+	94.3 (84.3–98.8)	61.0 (59.1–62.9) ***	4.6 (3.4–6.0)	99.8 (99.4–100.0)	1.02	0.89
5.HPV16/33/58/31+	94.3 (84.3–98.8)	55.6 (53.7–57.5) ***	4.0 (3.0–5.3)	99.8 (99.4–99.9)	1.02	0.81
6.HPV16/33/58/31/35+	94.3 (84.3–98.8)	53.3 (51.4–55.2) ***	3.8 (2.9–5.1)	99.8 (99.3–99.9)	1.02	0.78
*7.HPV16/18+ (comparator)*	92.5 (81.8–97.9)	68.7 (66.9–70.4)	5.5 (4.1–7.3)	99.8 (99.4–99.9)	1.00	1.00
8.HPV16/18/31/33/45/52/58+	98.1 (89.9–100.0)	31.6 (29.8–33.4) ***	2.8 (2.1–3.6)	99.9 (99.2–100.0)	1.06	0.46
**HPV types + or p16+**						
9.HPV16+ or p16+	98.1 (89.9–100.0)	62.8 (60.9–64.6) ***	5.0 (3.8–6.5)	99.9 (99.6–100.0)	1.06	0.91
10.HPV16/33+ or p16+	98.1 (89.9–100.0)	59.0 (57.1–60.9) ***	4.5 (3.4–5.9)	99.9 (99.6–100.0)	1.06	0.86
11.HPV16/33/58+ or p16+	100.0 (93.3–100.0)	49.5 (47.6–51.4) ***	3.8 (2.9–4.9)	100.0 (99.6–100.0)	1.08	0.72
12.HPV16/33/58/31+ or p16+	100.0 (93.3–100.0)	45.1 (43.2–47.1) ***	3.5 (2.6–4.6)	100.0 (99.6–100.0)	1.08	0.66
13.HPV16/33/58/31/35+ or p16+	100.0 (93.3–100.0)	43.4 (41.5–45.3) ***	3.4 (2.6–4.4)	100.0 (99.6–100.0)	1.08	0.63
14.HPV16/18+ or p16+	100.0 (93.3–100.0)	56.5 (54.6–58.3) ***	4.3 (3.3–5.7)	100.0 (99.7–100.0)	1.08	0.82
15.HPV16/18/31/33/45/52/58+ or p16+	100.0 (93.3–100.0)	27.0 (25.4–28.8) ***	2.6 (2.0–3.5)	100.0 (99.3–100.0)	1.08	0.39
**HPV types + & p16+**						
16.HPV16+ or HPV33/58/31/35 + &p16+ ^c^	92.5 (81.8–97.9) †	72.7 (71.0–74.4) ***†	6.3 (4.7–8.3)	99.8 (99.4–99.9)	1.00	1.06
17.HPV16/33+ or HPV58/31/35 + &p16+ ^c^	92.5 (81.8–97.9) †	68.9 (67.1–70.7) †††	5.6 (4.2–7.3)	99.8 (99.4–99.9)	1.00	1.00
18.HPV16/33/58/31/35 + &p16+	73.6 (59.7–84.7) *	85.4 (84.0–86.8) ***	9.1 (6.6–12.3)	99.4 (99.0–99.7)	0.80	1.24
19.HPV16/18/31/33/45/52/58 + &p16+ ^c^	77.4 (63.8–87.7) †	80.0 (78.5–81.5) ***†††	7.1 (5.2–9.6)	99.4 (99.0–99.7)	0.84	1.17

*Abbreviations*: *CI* confidence interval, *NPV* negative predictive value, *PPV* positive predictive value

^a^*P*-values for accuracy of the evaluated assay vs the ‘HPV16/18+’ strategy, * < 0.05, ** < 0.01, *** < 0.001

^b^Relative sensitivity or specificity of evaluated triage strategies relative to the ‘HPV16/18+’ triage

^c^*P*-values for accuracy of the evaluated assay vs the ‘HPV16/33+’ strategy, † > 0.05; †† < 0.01; ††† < 0.001

(i) Single test: HPV16+; HPV16/18+; HPV16/33+; HPV16/33/58+; HPV16/33/58/31+; HPV16/33/58/31/35+; HPV16/18/31/33/45/52/58+; p16 + .

(ii) HPV genotypes or p16 staining: HPV16/18+ or p16+; HPV16+ or p16+; HPV16/33+ or p16+; HPV16/33/58+ or p16+; HPV16/33/58/31+ or p16+; HPV16/33/58/31/35+ or p16+; HPV16/18/31/33/45/52/58+ or p16 + .

(iii) HPV genotypes & p16 staining: ‘HPV16/33+ or HPV58/31/35+&p16+’; ‘HPV16+ or HPV33/58/31/35+&p16+’; HPV16/33/58/31/35 + &p16+; HPV16/18/31/33/45/52/58 + &p16 + .

### Statistical analysis

CIN2+/CIN3+ were the main outcomes. For individual genotype, a hierarchical ordering was developed based on sequentially maximizing the positive predictive value (PPV) for the next HPV genotype after excluding women with multiple infections with types higher in the hierarchy [5, 26]. To define the optimal strategies, we considered a negative predictive value (NPV) of ≥98% for CIN3+, combined with improvement of sensitivity and specificity for CIN2+/CIN3+ relative to the comparator, being “HPV16/18+” strategy, to be the minimal requirements. Differences in sensitivity and specificity were evaluated with McNemar’s test. Relative sensitivity and specificity were determined and defined as the ratios of sensitivity and specificity between evaluated strategies and the comparator. To evaluate the clinical efficiency and the potential harms for the different algorithms, we calculated the number of referrals needed to detect one case (NRND) of CIN2+/CIN3+, the rates of colposcopy referral (CRR), the false positive rates (FPR = 1-specificity), and the false negative rates (FNR = 1-sensitivity) for the outcome of CIN2+, which is clinically relevant for treatment. Sensitivity and 1-specificity, along with respective 95%CIs, were plotted for each optimized strategy. Statistical analyses were conducted using SPSS 24.0. *P* < 0.05 was assumed to be statistically significant.

## Results

Overall, 5843 women were identified with HPV positivity. Those who had only lrHPV infection (*n* = 243), and those without p16 (*n* = 1402) or cytology result (*n =* 2) were excluded. Moving forward, those who had abnormal cytology (*n* = 1048) and those without pathologic results (*n* = 417) were excluded, leaving a pool of 2731 HPCN women included in the final analysis (Fig. 1). The mean age was 47.6 years (range: 30–64 years). Among them, 136(5.0%) cases of CIN2+ were identified, including 52(1.9%) CIN3 and one cancer.

Among the study population, the top five genotypes were HPV16 (23.7%), HPV52 (16.7%), HPV58 (14.0%), HPV51 (11.1%), and HPV18 (10.5%). The prevalence of multiple infections was 21.5% (588/2731). In the CIN2+ group, the most prevalent hrHPV genotypes were HPV16 (62.5%), HPV58(15.4%), HPV52(13.2%), HPV33(7.4%), HPV31(6.6%) and HPV18(6.6%); and 27.9% (38/136) of women were infected with multiple genotypes. The genotypes were divided into three strata (HPV16/33, HPV58/31/35, and other 9 types) according to their hierarchical risks for CIN2+ (Table S1).

**Fig. 2:**
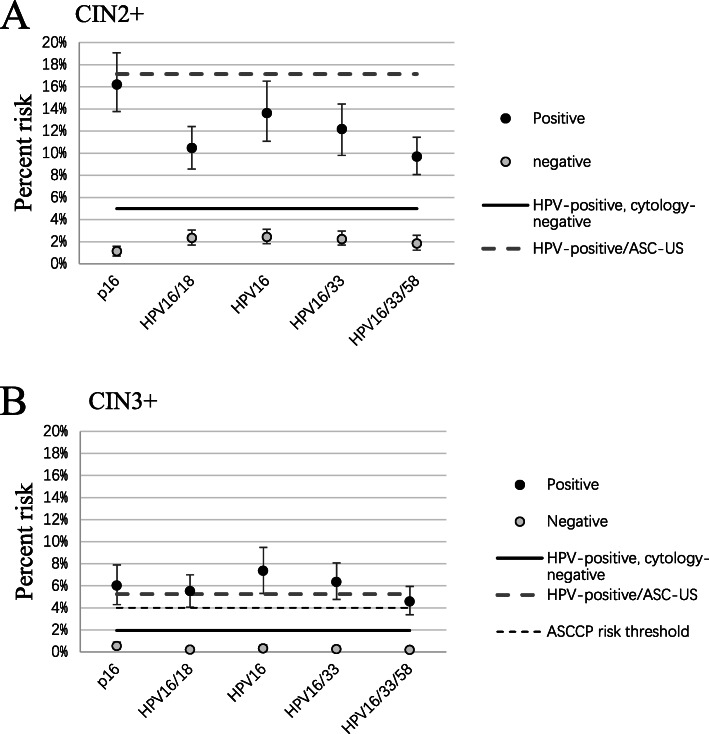
Percent risk of triage tests for CIN2+/CIN3+. ASC-US: atypical squamous cells of undetermined significance; CI, confidence interval; CIN2+/CIN3+: cervical intraepithelial neoplasia 2/3 or worse; ASCCP: the American Society of Colposcopy and Cervical Pathology. (**a**) For CIN2+, boxes with vertical lines indicate point estimates of risk with 95% CIs. The dashed line indicates risk of HPV-positive ASC-US, which is 17.1% in this population. The solid line indicates risk for HPV positive/cytology negative result, which is 5.0%. (**b**) For CIN3+, boxes with vertical lines indicate point estimates of risk with 95% CIs, the thick dashed line indicates risk of HPV-positive ASC-US, which is 5.2% in this population. The solid line indicates risk for HPV positive/cytology negative, which is 1.9%. The thin dashed line indicates the ASCCP risk threshold (4%). All these tests showed in the figure reached the ASCCP risk threshold for immediate colposcopy referral

The p16 positivity rates among three genotype strata were 40.2%(HPV16/33), 22.9% (HPV58/31/35), and 18.8% (other 9 types) respectively, increasing with the elevation of hierarchical types (*P* < 0.0001; Table S2). Interestingly, p16 staining could further stratify the risk of HPV16/33, HPV58/31/35 and the rest of 9 types. Importantly, five triage tests (p16, HPV16, HPV16/18, HPV16/33, and HPV16/33/58) showed fine risk stratification both for CIN2+/CIN3 + (Fig. 2). The overall risks of HPV+/ASC-US result (10.2 and 5.2% respectively) for CIN2+/CIN3+ were taken as the benchmark for immediate colposcopy referral previously [6]. The risks of positive result for p16, or HPV16, or HPV16/18, or HPV16/33 surpassed the benchmark risk for CIN3+, although not for CIN2+ (Fig. 2). Moreover, the risks of positive result for all tests in Fig. 2 were above the 2019 ASCCP risk threshold (4%) for colposcopy referral [12].

**Fig. 3:**
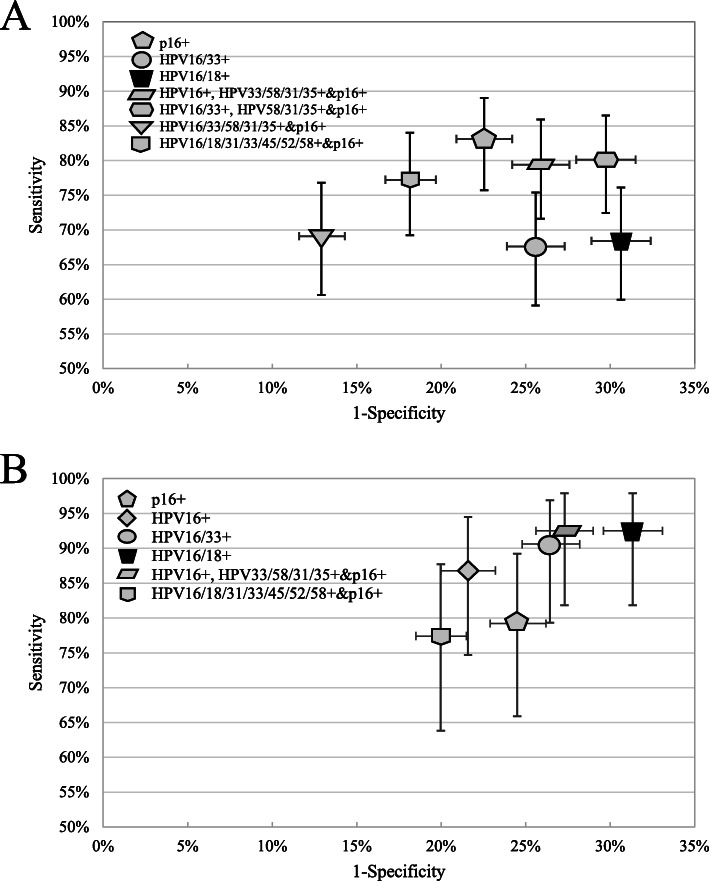
Sensitivity and 1-specificity of optimized triage strategies in hrHPV-positive, cytology-negative women. CIN2+/CIN3+: cervical intraepithelial neoplasia 2/3 or worse. (A) CIN2+, *n =* 136; (B) CIN3+, *n* = 53. Bars represented 95% confidence intervals. The black trapezoid represents HPV16/18 genotyping. The favorable strategies to HPV16/18 genotyping are shown in gray

***Strategy 7*** (HPV16/18+) showed a sensitivity of 92.5% (81.8–97.9%) and a specificity of 68.7% (66.9–70.4%) for CIN3+ (Table 2), and was used as the comparator. All the 19 triage strategies met the test criterion of an NPV of ≥98% for CIN3+. However, only five strategies showed a notable improvement in specificity and/or sensitivity for CIN3+/CIN2+ to the comparator: **Strategy 1** (p16+); **Strategy 3 (**HPV16/33+); **Strategy 16** (HPV16+ or HPV33/58/31/35 + &p16+); **Strategy 17** (HPV16/33+ or HPV58/31/35 + &p16+); **Strategy 15** (HPV16/18/31/33/45/52/58 + &p16+), see Tables 1 & 2, and Fig. 3. Of these strategies, **Strategy 1** detected 83.1% (79.2%) of underlying CIN2+ (CIN3+) among HPCN women. **Strategy 1 and 16** were favorable in specificity and sensitivity for CIN2+/CIN3+. **Strategy 3** and **Strategy 15** revealed a favorable specificity without a reduction in sensitivity. In addition, **Strategy 17** was favorable in sensitivity and similar in specificity for detecting CIN2+/CIN3+ to the comparator (Tables 1 & 2, Fig. 3). **Strategy 2** (HPV16+) showed a lower sensitivity (relative sensitivity = 0.91, *P* = 0.008) for CIN2+ to the comparator. **Strategy 18** (HPV16/33/58/31/35+ &p16+) was less sensitive to the comparator for CIN3+ (relative sensitivity = 0.80, *P* = 0.013). The other strategies showed an improved or comparable sensitivity for detecting CIN2+/CIN3+ at the expense of a lower specificity to the comparator (Tables 1 & 2).

To investigate the relative accuracy of the strategies incorporating p16 to strategy incorporating HPV genotyping alone among the optimal strategies, we further compared the **Strategy 3** (HPV16/33+) to the other four strategies, and found that the **Strategy 1**, **Strategy 16** and **Strategy 15** were favorable in sensitivity and/or specificity to **Strategy 3**. Whereas the **Strategy 17** had a higher sensitivity but a lower specificity than **Strategy 3** for detecting CIN2+ (Tables 1 & 2).

Triaging HPCN women with ***Strategy 7*** (HPV16/18+) required a CRR of 32.5%, and NRNDs of 9.5/18.1 for CIN2+/CIN3+, resulting in an FPR/FNR of 30.6%/31.6% for CIN2+ (Table 3). ***Strategy 17*** required a similar CRR/FPR of 32.3%/29.7%, and a comparable NRND (18.0) for CIN3+, but a less NRND (8.1 vs 9.5) for CIN2+, leading to a better FNR of 19.9% for CIN2+ to the comparator. Triage with the other four optimal strategies above (**Strategy 1, 3, 15, 16)** provided lower CRRs (range 21.1–28.6% vs 32.3%, *P* < 0.05), less NRNDs (for CIN2+, range 5.5–8.2 vs 9.5; for CIN3+, range 14.0–16.6 vs 18.1), accompanied by better FPRs (range 18.2–25.9% vs 30.6%) and better FNRs than ***Strategy 7*** for CIN2+ (the ‘HPV16/33+’: 32.4%; Other three strategies: range 16.9–22.8% vs 31.6%) (Table 3).

**Table 3:** Disease detection, colposcopies needed, and false positive (negative) rates of different triage strategies (*N* = 2731)

Triage strategies	Number of colposcopies	CRR (%)	Number of CIN2+/ CIN3+ detected	NRND for CIN2+/CIN3+	FPR (%) for CIN2+	FNR (%) for CIN2+
**Single test**						
1.p16+	698	25.6	113/42	6.2/16.6	22.5	16.9
2.HPV16+	624	22.8	85/46	7.3/13.6	20.8	37.5
3.HPV16/33+	756	27.7	92/48	8.2/15.8	25.6	32.4
4.HPV16/33/58+	1094	40.1	106/50	10.3/21.9	38.1	22.1
5.HPV16/33/58/31+	1240	45.4	111/50	11.2/24.8	43.5	18.4
6.HPV16/33/58/31/35+	1301	47.6	113/50	11.5/26.0	45.8	16.9
*7.HPV16/18 + (comparator)*	888	32.5	93/49	9.5/18.1	30.6	31.6
8.HPV16/18/31/33/45/52/58+	1885	69.0	125/52	15.1/36.3	67.8	8.1
**HPV types + or p16+**						
9.HPV16+ or p16+	1049	38.4	127/52	8.3/20.2	35.5	6.6
10.HPV16/33+ or p16+	1150	42.1	128/52	9.0/22.1	39.4	5.9
11.HPV16/33/58+ or p16+	1406	51.5	132/53	10.7/26.5	49.1	2.9
12.HPV16/33/58/31+ or p16+	1522	55.7	132/53	11.5/28.7	53.6	2.9
13.HPV16/33/58/31/35+ or p16+	1570	57.5	132/53	11.9/29.6	55.4	2.9
14.HPV16/18+ or p16+	1219	44.6	129/53	9.4/23.0	42.0	5.1
15.HPV16/18/31/33/45/52/58+ or p16+	2007	73.5	133/53	15.1/37.9	72.2	2.2
**HPV types + & p16+**						
16.HPV16+ or HPV33/58/31/35 + &p16+	780	28.6	108/49	7.2/15.9	25.9	20.6
17.HPV16/33+ or HPV58/31/35 + &p16+	881	32.3	109/49	8.1/18.0	29.7	19.9
18.HPV16/33/58/31/35 + &p16+	429	15.7	94/39	4.6/11.0	12.9	30.9
19.HPV16/18/31/33/45/52/58 + &p16+	576	21.1	105/41	5.5/14.0	18.2	22.8

*CRR* Colposcopy referral rate, *NRND* The number of referrals needed to detect one case, *FPR* False positive rate = 1-specificity, proportion of index test positives among histologic normal results (cervical intraepithelial neoplasia grade 1 or less), *FNR* False negative rate = 1-sensitivity, proportion of index test negatives among histologic abnormal results (CIN2+)

## Discussion

Dodd et al. reported that HPV-positive women showed higher anxiety scores, and expressed greater distress about their test result and cancer worry than HPV-negative women [27]. Additionally, the prevalence of clinically relevant CIN2+ among HPCN women varies significantly across HPV types [9, 16, 26], settings and different populations, with 5.0% in our study from a general population, and about 20% from a outpatient population [26]. Therefore, quite a few cervical high-grade lesions would be undiagnosed by LBC in HPV-positive women. Optimal risk-based approach is urgently needed to identify HPCN women deemed at greatest risk of clinically relevant CIN2+ for colposcopy, while delaying further investigations among those in whom HPV infection is most likely to clear.

Currently, there still lacks an ideal triage option and associated evidences for HPV-positive self-samples. Cytology is a common triage method, the optimal management of HPCN women is controversial. Thus, the performance of multiple strategies based on extended genotyping and p16^INK4a^ immunostaining, alone or combined, was assessed for the triage of HPCN women, in comparison with HPV16/18 genotyping. Surprisingly, five triage strategies showed a significant improvement in specificity and/or sensitivity for CIN2+/CIN3+ to the comparator: 1) p16+; 2) HPV16/33+; 3) HPV16+ or HPV33/58/31/35 + &p16+; 4) HPV16/33+ or HPV58/31/35 + &p16+; 5) HPV16/18/31/33/45/ 52/58+ &p16+. The high NPV and improved sensitivity and/or specificity of these strategies would safely allow less frequent repetitions of screening for low-risk women [28], but also avoid substantial unnecessary colposcopies, thus substantially improve the workload of healthcare services and potential mental burden of the women involved.

A recent meta-analysis showed that an HPCN women had on average a 6.4% risk of CIN3+ within 5 years, but her 1-year risk of CIN3+ was less than 4%, which was proposed as the threshold for immediate referral to colposcopy in the most recent ASCCP guidelines [9]. Similarly, our study calculated an immediate risk of 1.9% for CIN3+ in HPCN women. They should be referred to one-year retest according to the US guidelines [12]. Unfortunately, this method is only suitable for screening programs with high adherence to early recall. Their follow-up will substantially influence screening costs and workload, potentially leading to mental burden for women involved and the loss to follow-up [29], which is a principal problem in low-resource areas and results in the missed diagnosis of existing CIN2+. Thus, more accurate interventions are needed to increase the effectiveness of the screening in HPCN women, particularly for those with lower attendance [29].

Interestingly, HPV assays provide individual genotype information help clarify the type-specific risks [6, 13, 15], and incorporating the most carcinogenic types is useful and may be efficient for the triage of HPCN women. HPCN women infected with HPV16/18 and other hrHPV had 3-year risks of 15.5 and 3.1% respectively for CIN2+ [8], and 5-year risks of 18.1 and 3.5% respectively for CIN3+ [30]. Due to the high oncogenicity of HPV16/18, previous studies largely focused on these two types [31, 32], and rare studies elucidate the role of extended genotyping in the triage of HPV-positive women [33]. Thus, the extent to which adding other hrHPV-types in the triage of HPCN women could improve lesion detection, remains unclear and needs to be determined. In this study HPV33 conferred a high-risk only next to HPV16, which was found in several previous studies [6, 16, 34]. Moreover, HPV16/33 as triage effectively reduced the colposcopy referral while maintaining similar diagnostic efficacy to HPV16/18 as triage. Importantly, genotypes have an obvious advantage of being objective and reproducible since they are automatically provided, thus are practical and economical.

However, we furtherly found that strategies incorporating p16 staining could achieve a preferable accuracy either alone or combined with genotypes than strategies derived from genotyping alone (the ‘HPV16/18+’ and the ‘HPV16/33+’) in screening. The reasons may be that management by genotyping alone is based solely on risk of cervical precancer or cancer without knowledge about what’s occurring within the cells [33]; it’s likely to miss the diagnosis for women who have infected certain genotype associated with a lower risk but that is already transforming into a cancerous disease; remarkably, p16 immunostaining is an indicator of malignant transformation of HPV infection and provides genotype agnostic risk information. Besides, the combinations of genotypes and p16 immunostaining would reduce the cost of p16 assay compared to p16 immunostaining alone as triage.

Although the utility of p16 staining may somewhat increase the costs of screening tests relative to pure genotyping as the triage, it could improve the colposcopy referral and reduce the short-term follow-up burden, leading to the early detection of cervical precancers, therefore it may be a cost-effective and efficient strategy in the long run. Moreover, due to the low-cost of HPV genotyping assay and the high-throughput and easy-to-interpret results of p16 staining utilized in our study, they are affordable and likely to serve as a potential candidate for large cervical screening programs [23]. Notably, as costs are area specific, a full economic evaluation and resource availability are warranted to consider when incorporating these optimized strategies into practice.

p16/Ki-67 dual staining might improve the specificity of p16 staining and attracts attention recently [32]. A previous study on p16/Ki-67 triage of HPCN result showed that p16/Ki-67 detected more than 70% of underlying CIN3+ lesions at baseline, while specificity for CIN3+ was significantly lower for p16/Ki-67 versus HPV16/18 genotyping (70.0% vs. 78.3%, *p* = 0.005) [35]. Herein, p16 immunostaining significantly improved the sensitivity and specificity relative to genotyping for HPV16/18 as triage. Currently it’s hard to draw the conclusion that p16/Ki-67 is better than p16 staining in terms of the diagnostic performance. Further investigations are needed to explore both methods in the same population.

Although several previous studies have analyzed the role of p16 immunostaining in the early detection of cervical cancer [36], few robust, exploratory studies were focused on the management of HPCN women. This is to date the first and largest study to investigate the performance of p16 immunostaining detected by a newly developed technology, PathCIN®p16^INK4a^ kits, in triaging HPCN women. In addition, recently several studies have analyzed genotype distribution and type-specific risks in HPCN women in China [16, 26], to our knowledge, none of them have directly evaluated the accuracy performance of extended genotyping, especially in combination with p16 staining, in triaging HPCN women. Moreover, there are several strengths to this study. Firstly, this large-size, population-based study contains high-quality data on results of HPV genotypes, cytology, colposcopy and accurate disease ascertainment with quality control. The robust results can be extended to general population. Strikingly, a sample-size of 2731 HPCN women achieves 99.7% power to detect a difference of 0.018 using a two-sided exact test with a significance level (alpha) of 0.05; these results assume that the population proportion under the null hypothesis is 3.2% per the result of CHIMUST trial [7]. Thus the large size permitted a strong statistical power. Secondly, due to the prospective nature of this study, the number of technically invalid samples for the p16 staining low to nonexistent. One limitation of our study was the fact that LBC testing was based on residual liquid after p16 immunostaining. In addition, as a cross-sectional study, the safe interval cannot be predicted for negative test result, and the natural history of HPCN or cervical lesions is unknown since some infections or lesions will regress or progress. Long-term follow-up data on HPCN women are expected furtherly.

## Conclusions

In summary, genotyping for HPV16/33 could be utilized to optimize the management of HPCN women. Moreover, p16 immunostaining, either alone or combined with extended genotypes, is more effective than HPV genotypes alone in the triage of HPCN women. Hopefully, these findings provide new insights into optimizing the management of HPCN women, and offer a potent evidence for the implementation of HPV-based screening on self-samples.

## Supplementary Information

**Additional file 1: Table S1.** Positive Predictive Value (PPV) for CIN2+ and CIN3+. **Table S2.** p16 positivity stratified by HPV genotypes and histologic grades (n, %). **Fig. S1**. Management protocol of HPV-positive women. VIA, visual inspection under acetic acid; LBC, liquid-based cytology. Genotyping for HPV16/18, VIA, and p16 immunostaining were used for triage sequentially. **Fig. S2.** p16 immunostaining, (A) positive, one or more cervical epithelial cells with nuclear and/or cytoplasmic stained brownish or yellow were defined as positive regardless of their morphology. (B) negative.

## Data Availability

The datasets generated and/or analyzed during the current study are not publicly available but are available from the corresponding author on reasonable request.

## Abbreviations

ASC-US: Atypical squamous cells of undetermined significance
LBC: Liquid-based cytology
HPCN: HPV-positive, cytology-negative
PPV: Positive predictive value
NPV: Negative predictive value
CRR: Colposcopies referral rate
NRND: Number of referrals needed to detect one lesion
FPR: False positive rate
FNR: False negative rate
