# Supplementary material for: Triaging HPV-positive, cytology-negative cervical cancer screening results with extended HPV genotyping and p16INK4a immunostaining in China
Source: BMC Infect Dis. 2021 Apr 30;21:400. doi: 10.1186/s12879-021-06109-4 (PMC8086315; doi:10.1186/s12879-021-06109-4)
Supplement: Supplementary file 1 — Additional file 1: Table S1. Positive Predictive Value (PPV) for CIN2+ and CIN3+. Table S2. p16 positivity stratified by HPV genotypes and histologic grades (n, %). Fig. S1. Management protocol of HPV-positive women. VIA, visual inspection under acetic acid; LBC, liquid-based cytology. Genotyping for HPV16/18, VIA, and p16 immunostaining were used for triage sequentially. Fig. S2. p16 immunostaining, (A) positive, one or more cervical epithelial cells with nuclear and/or cytoplasmic stained brownish or yellow were defined as positive regardless of their morphology. (B) negative. [file 12879_2021_6109_MOESM1_ESM.docx]

**Triaging HPV-positive, cytology-negative cervical cancer screening results with extended HPV genotyping and p16^INK4a^ immunostaining in China**

Fangbin Song^1,2†^, Peisha Yan^1,2†^, Xia Huang^1,2^, Chun Wang^1,2^, Xinfeng Qu^3^, Hui Du^1,2*^ and Ruifang Wu^1,2*^
^1^Department of Obstetrics and Gynecology, Peking University Shenzhen Hospital, Shenzhen, Guangdong, PR China.

^2^Shenzhen Key Laboratory on Technology for Early Diagnosis of Major Gynecological Diseases, Shenzhen, Guangdong, PR China.

^3^Sanming Project of Medicine in Shenzhen, Peking University Shenzhen Hospital, 518036 Shenzhen, Guangdong, PR China.

*****Correspondence to**:

Ruifang Wu, Department of Obstetrics and Gynecology, Peking University Shenzhen Hospital, No. 1120 Lianhua Road, 518036 Shenzhen, Guangdong, PR China. E-mail: wurfpush@126.com.

Hui Du, Department of Obstetrics and Gynecology, Peking University Shenzhen Hospital, No. 1120 Lianhua Road, 518036 Shenzhen, Guangdong, PR China. E-mail: duhui_107108@163.com.

**Table S1. Positive Predictive Value (PPV) for CIN2+ and CIN3+**

| **Diagnostic Test** |  | **CIN2+** | | | **CIN3+** | |
| --- | --- | --- | --- | --- | --- | --- |
|  | **Total** | **N** | **PPV ^a^** | **Classification ^b^** | **N** | **PPV** |
| **p16** |  |  |  |  |  |  |
| Positive | 698 | 113 | 16.2% |  | 42 | 6.0% |
| negative | 2033 | 23 | 1.1% |  | 11 | 0.5% |
| **Genotypes** |  |  |  |  |  |  |
| **HPV16** | 624 | 85 | 13.6% | A | 46 | 7.4% |
| **HPV33** | 132 | 7 | 5.3% | A | 2 | 1.5% |
| **HPV58** | 338 | 14 | 4.1% | B | 3 | 1.2% |
| **HPV31** | 146 | 5 | 3.4% | B | 1 | 0.7% |
| **HPV35** | 61 | 2 | 3.3% | B | 1 | 0.3% |
| HPV66 | 122 | 4 | 3.3% | C | 0 | 0.0% |
| HPV18 | 222 | 6 | 2.7% | C | 0 | 0.0% |
| HPV52 | 375 | 8 | 2.1% | C | 0 | 0.0% |
| HPV56 | 96 | 2 | 2.1% | C | 0 | 0.0% |
| HPV68 | 171 | 2 | 1.2% | C | 0 | 0.0% |
| HPV51 | 191 | 1 | 0.5% | C | 0 | 0.0% |
| HPV39 | 131 | 0 | 0.0% | C | 0 | 0.0% |
| HPV45 | 38 | 0 | 0.0% | C | 0 | 0.0% |
| HPV59 | 84 | 0 | 0.0% | C | 0 | 0.0% |
| Total | 2731 | 136 | 5.0% | C | 53 | 1.9% |

PPV, positive predictive value.

^a^ For new genotype omitting multiple infections with types higher in the HPV genotype hierarchy.

^b^ HPV genotypes were classified as three levels (A/B/C) according to the hierarchy ranking for CIN2+. Type group A/B were included into the triage strategies for analysis.

**Table S2. p16 positivity stratified by HPV genotypes and histologic grades (n, %)**

| **Variables** | **p16** | **Normal** | **CIN1** | **CIN2** | **CIN3** | **Cancer** | **Total** |
| --- | --- | --- | --- | --- | --- | --- | --- |
| hrHPV infection | Total | 2388 | 207 | 83 | 52 | 1 | 2731 |
|  | Positive | 478 (20.0) | 107 (51.7) | 71 (85.5) | 41 (78.8) | 1 (100.0) | 698 (25.6) |
|  | Negative | 1910 (80.0） | 100 (48.3) | 12 (14.5) | 11 (21.2) | 0 (0.0) | 2033 (74.4) |
| HPV16/18 | Total | 694 | 101 | 44 | 48 | 1 | 888 |
|  | Positive | 241 (34.7) | 49 (48.5) | 39 (88.6) | 37 (77.1) | 1 (100.0) | 367 (41.3) |
|  | Negative | 453 (65.3) | 52 (51.5) | 5 (11.4) | 11 (22.9) | 0 (0.0) | 521 (58.7) |
| Other 12 types | Total | 1694 | 106 | 39 | 4 | 0 | 1843 |
|  | Positive | 237 (14.0) | 58 (54.7) | 32 (82.1) | 4 (100.0) | 0 (0.0) | 331 (18.0) |
|  | Negative | 1457 (86.0) | 48 (45.3) | 7 (17.9) | 0 (0.0) | 0 (0.0) | 1512 (82.0) |
| HPV16/33 | Total | 581 | 83 | 44 | 47 | 1 | 756 |
|  | Positive | 186 (32.0) | 41 (49.4) | 39 (88.6) | 37 (78.7) | 1 (100.0) | 304 (40.2） |
|  | Negative | 395 (68.0) | 42 (50.6) | 5 (11.4) | 10 (21.3) | 0 (0.0) | 452 (59.8) |
| HPV58/31/35 | Total | 485 | 39 | 19 | 2 | 0 | 545 |
|  | Positive | 88 (18.1) | 20 (51.3) | 16 (84.2) | 1 (50.0) | 0 (0.0) | 125 (22.9) |
|  | Negative | 397 (81.9) | 19 (48.7) | 3 (15.8) | 1 (50.0) | 0 (0.0) | 420 (77.1) |
| Other 9 types | Total | 1322 | 85 | 20 | 3 | 0 | 1430 |
|  | Positive | 204 (15.4) | 46 (54.1) | 16 (80.0) | 3 (100.0) | 0 (0.0) | 269 (18.8) |
|  | Negative | 1118 (84.6) | 39 (45.9) | 4 (20.0) | 0 (0.0) | 0 (0.0) | 1161 (81.2) |


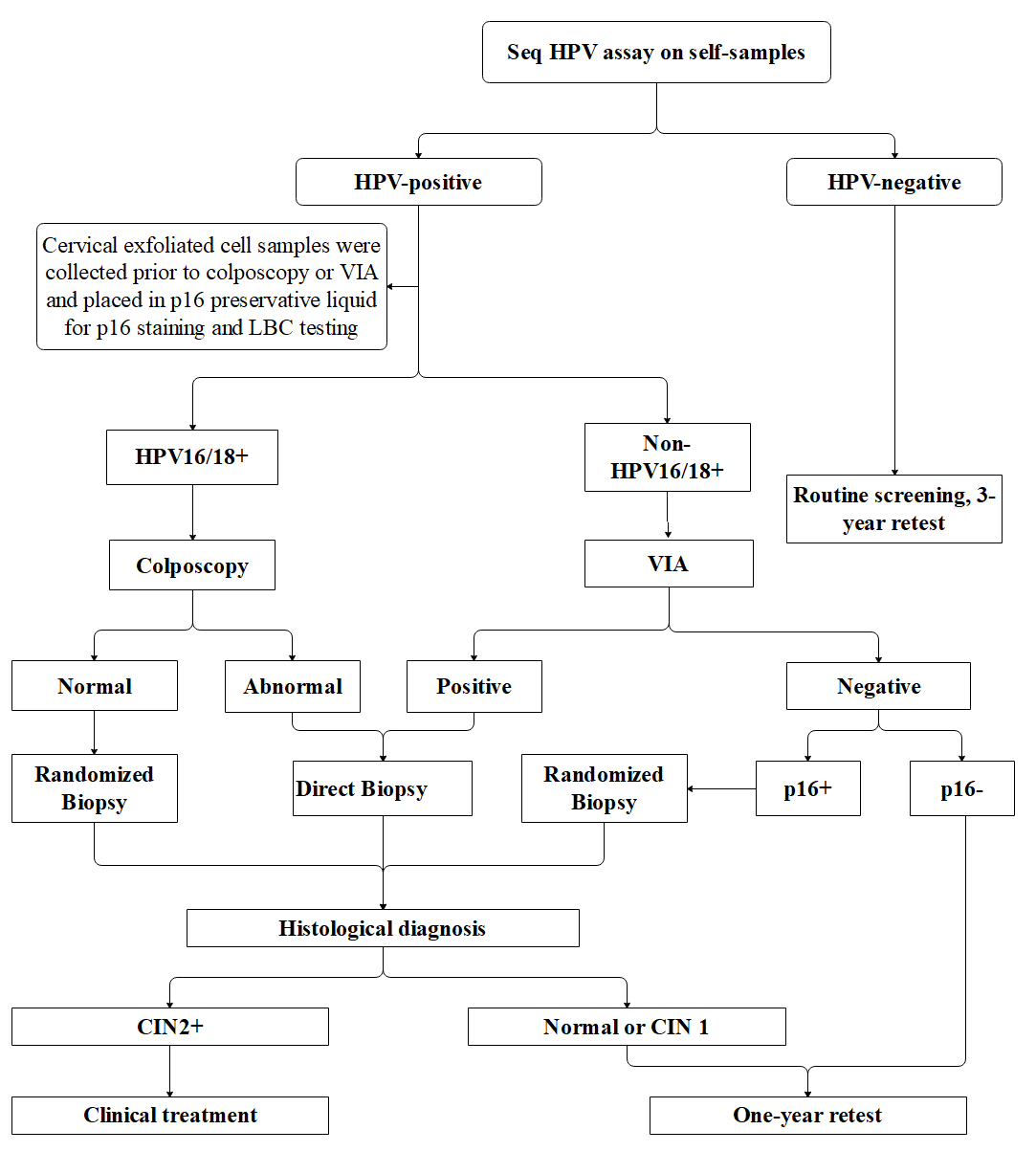


**Figure S1.** Management protocol of HPV-positive women. VIA, visual inspection under acetic acid; LBC, liquid-based cytology. Genotyping for HPV16/18, VIA, and p16 immunostaining were used for triage sequentially.


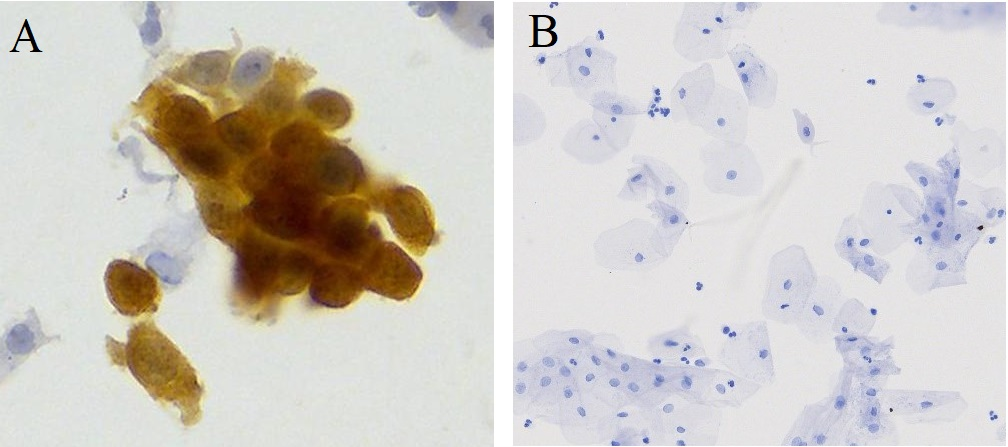


**Figure S2.** p16 immunostaining, (A) positive, one or more cervical epithelial cells with nuclear and/or cytoplasmic stained brownish or yellow were defined as positive regardless of their morphology. (B) negative.
